# Revisiting the morbid genome of Mendelian disorders

**DOI:** 10.1186/s13059-016-1102-1

**Published:** 2016-11-24

**Authors:** Mohamed Abouelhoda, Tariq Faquih, Mohamed El-Kalioby, Fowzan S. Alkuraya

**Affiliations:** 1 Department of Genetics, King Faisal Specialist Hospital and Research Center, Riyadh, Saudi Arabia; 2 Saudi Human Genome Program, King Abdulaziz City for Science and Technology (KACST), Riyadh, Saudi Arabia; 3 Department of Anatomy and Cell Biology, College of Medicine, Alfaisal University, Riyadh, Saudi Arabia

**Keywords:** Saudi Human Genome Program (SHGP), Autozygosity, Variant classification, Disease mutations

## Abstract

**Background:**

The pathogenicity of many Mendelian variants has been challenged by large-scale sequencing efforts. However, many rare and benign “disease mutations” are difficult to analyze due to their rarity. The Saudi Arabian variome is enriched for homozygosity due to inbreeding, a key advantage that can be exploited for the critical examination of previously published variants.

**Results:**

We collated all “disease-related mutations” listed in the Human Gene Mutation Database (HGMD) and ClinVar, including “variants of uncertain significance” (VOUS). We find that the use of public databases including 1000 Genomes, ExAC, and Kaviar can reclassify many of these variants as likely benign. Our Saudi Human Genome Program (SHGP) can reclassify many variants that are rare in public databases. Furthermore, SGPD allows us to observe many previously reported variants in the homozygous state and our extensive phenotyping of participants makes it possible to demonstrate the lack of phenotype for these variants, thus challenging their pathogenicity despite their rarity. We also find that 18 VOUS *BRCA1* and *BRCA2* variants that are listed in BRCA Exchange are present at least once in the homozygous state in patients who lack features of Fanconi anemia. Reassuringly, we could reciprocally demonstrate that none of those labeled as “pathogenic” were observed in the homozygous statue in individuals who lack Fanconi phenotype in our database.

**Conclusion:**

Our study shows the importance of revisiting disease-related databases using public resources as well as of population-specific resources to improve the specificity of the morbid genome of Mendelian diseases in humans.

**Electronic supplementary material:**

The online version of this article (doi:10.1186/s13059-016-1102-1) contains supplementary material, which is available to authorized users.

## Background

DNA variation in human populations express itself phenotypically in an extremely broad spectrum ranging from embryonic lethality to complete lack of phenotypes [1–5]. An added layer of complexity lies in the effect size of the variants, which again displays a wide range from the infinitesimal to the fully penetrant. Historically, study of DNA variants has focused on those that are highly penetrant with a recognizable clinical consequence, so-called Mendelian variants [6]. Despite the recent explosion in our knowledge of how variants can influence risk for many common diseases, Mendelian variants remain the most clinically actionable, with most genetically informed decisions about reproductive choices and medical management being linked to this class of variants [6].

Unlike most laboratory tests in the clinical setting, medical DNA sequencing is qualitative in nature and requires extensive assessment of each variant prior to reporting. Until recently, there were no standard guidelines for establishing disease-variant links. The commonly held view that truncating variants are pathogenic was first challenged by the finding that many single nucleotide polymorphisms (SNPs) are truncating in nature and this was further confirmed by large-scale sequencing studies [1, 7–10]. Similarly, the practice of screening 100 individuals of a similar ethnicity to confirm pathogenicity of a given allele suffered the fallacy of the 1% frequency definition of SNPs, which is now seen as arbitrary since there are many rare, ultra-rare, or even private SNPs that do not cause any Mendelian disease. This has resulted in the publication of numerous variants that have subsequently been uploaded to major databases as “disease mutations” with little evidence of pathogenicity [11, 12].

The recent American College of Medical Genetics and Genomics (ACMG) guidelines on the interpretation of variants provide the much-needed standardization of reporting variants, although labs continue to have conflicting interpretation even when using these guidelines [13, 14]. Key in these guidelines is the emphasis on allele frequency and segregation with the phenotype [14]. Alleles with a frequency that is too high for the disease in question and those that do not segregate with the phenotype should be classified as benign, the issue of reduced penetrance notwithstanding. Indeed, many “disease mutations” were subsequently found to be benign using large-scale sequencing efforts [12]. Similarly, the increasingly common use of clinical genomics has revealed many “disease mutations” that clearly do not segregate with the disease, although efforts to make such valuable findings publicly available remain limited [15].

In this study, we hypothesized that there are false “disease mutations” whose frequency in 1000 Genome, Kaviar, and ExAC is low but are in fact common variants among Saudi Arabians, an ethnicity with poor representation in the aforementioned variant databases and paradoxically high contribution to “disease mutations” based on positional mapping studies. We also hypothesized that the high degree of inbreeding among Saudi Arabians will render homozygous at least some “disease mutations” in individuals who lack the phenotype. In both instances, large-scale sequencing of Saudi Arabians should permit the reclassification of many alleles as benign, which is otherwise not possible using 1000 Genomes, Kaviar, and ExAC, as we demonstrate in this study.

## Results

### The morbid genome has specificity limitations

As of August 2016, there are 187,319 variants listed in HGMD related to “disease mutations” (Classes, DM, “DM?”), mostly missense (76%). The corresponding ClinVar list comprises 82,543 variants, including 37,345 pathogenic, 7914 likely pathogenic, and 37,284 VOUS, mostly missense (86%).

Table 1 summarizes the results of reclassifying the variants in the Human Gene Mutation Database (HGMD) and ClinVar based on the four criteria described in “Methods” (all reclassified variants are included in the supplemental tables). The number of HGMD disease-related mutations, including classes DM and DM?, that can be classified as benign based on population frequency and their presence in disease-free individuals as inferred from 1000 Genomes, Kaviar, and ExAC (see “Methods”) was 255 at an allele frequency threshold of 5% (Additional file 1: Table S1).

**Table 1:** Number of reclassified variants as per each criterion in both HGMD and ClinVar

HGMD				
Reclassification criteria	DM (167,938 variants)	DM? (19,381 variants)		Total reclassified
Criteria1: > = 0.05 (Additional file 1: Table S1)	22	233		255
Criteria 2: > = 0.05 in SGP (and < 0.05 in PublicDB) (Additional file 3: Table S3)	1	15		16
Criteria 3: > = 0.01 in SGP (at least 1 hom) (Additional file 5: Table S5)	103	504		607
Criteria 4: <0.01, hom in SGP, no association with disease (Additional file 7: Table S7)	107	108		215
				Total = 1093
ClinVar				
Reclassification criteria	Pathogenic (37,345 variants)	Likely pathogenic (7914 variants)	Uncertain significance (37,284 variants)	Total reclassified
Criteria1 > = 0.05 (Additional file 2: Table S2)	122	18	194	334
Criteria 2: > = 0.05 in SGP (and < 0.05 in PublicDB) (Additional file 4: Table S4)	6	0	4	10
Criteria 3: > = 0.01 in SGP (at least 1 hom) (Additional file 6: Table S6)	184	25	275	484
Criteria 4: <0.01, hom in SGP, no association with disease (Additional file 8: Table S8)	21	6	43	70
				Total = 898

Reclassified variants based on our four exclusion criteria for the HGMD and ClinVar databases. The numbers between brackets for Criteria 1 are for the number of variants filtered by 1000 Genomes, then by ExAc, then by Kaviar

As expected based on its more recent development, its reliance on large public variant databases, and more stringent scoring system, the number of variants listed by ClinVar that could be classified as benign was much smaller: 122 pathogenic and 18 likely pathogenic at an allele frequency threshold of 5% (Additional file 2: Table S2). Similarly, the number of ClinVar VOUS that can be reclassified as benign based on 1000 Genomes, Kaviar, or ExAC was 194 at an allele frequency threshold of 5% (Additional file 2: Table S2). Reassuringly, very few of the “reclassified” variants in ClinVar and HGMD were truncating in nature (8.0% [7.9% HGMD, 8.1% ClinVar]).

### The Saudi Human Genome Program database can improve the annotation of the human morbid genome

Because many disease genes were first described in Arab patients given the high rate of consanguinity, which facilitates positional mapping, and because this ethnicity is underrepresented in publicly available variant databases, we expected that at least some published “disease variants” may represent Arab-specific or Arab-enriched common variants that cannot be identified as such using these databases. On the other hand, the Saudi Human Genome Program (SHGP) database, which represents the largest database of genetic variants from individuals of Arab ethnicity, should uncover such Arab-specific and Arab-enriched common variants. Indeed, our analysis revealed that 16 variants in HGMD (1 DM and 15 DM?) and 10 in ClinVar (6 Pathogenic, 4 VOUS) achieved a population frequency of 5% in the SHGP database but were <5% in public databases so they can be confidently reclassified as benign (Additional file 3: Table S3 and Additional file 4: Table S4). Furthermore, because the SHGP database is enriched for autozygosity, we were able to reclassify an additional 607 HGMD (103 DM and 504 DM?) variants and 484 ClinVar (184 Pathogenic, 25 Likely Pathogenic, 275 VOUS) variants as benign based on a population frequency of >1% and their presence in the homozygous state in individuals who lack the reported phenotype (Additional file 5: Table S5 and Additional file 6: Table S6). A special scenario was frequently encountered when an allele only met the BS2 criterion of the ACMG guidelines (i.e. presence in homozygosity in disease-free individuals) but not BS1 (i.e. frequency too high for the known disease frequency). Such variants cannot be reclassified as benign or even likely benign without additional lines of evidence according to the ACMG guidelines. However, we believe knowledge of the existence of these variants in homozygosity in disease-free individuals can be very informative in the interpretation of these variants even though they did not achieve a population frequency of 1%. Therefore, we opted to designate these alleles as “BS2-only variants.” As expected by the high level of autozygosity in the SHGP database, we have encountered 215 HGMD (107 DM and 108 DM?) and 70 ClinVar (21 Pathogenic, 43 VOUS, 6 Likely Pathogenic) variants in homozygosity in individuals who lack the reported phenotype and these are listed as BS2-only variants (Additional file 7: Table S7 and Additional file 8: Table S8).

### The SHGP database and the BRCA challenge

As listed on their website, “The BRCA Challenge of the Global Alliance for Genomics and Health aims to advance understanding of the genetic basis of breast cancer and other cancers by pooling data on BRCA genetic variants from around the world, bringing together information on sequence variation, phenotype and scientific evidence.” After downloading all pathogenic and VOUS variants listed in their data repository (BRCA Exchange), we interrogated these variants for frequency in the SHGP Database and found 16 variants that have MAF > 0.01. Five of these were observed at least once in homozygosity in individuals who lack the Fanconi anemia phenotype thus can be confidently classified as benign. On the other hand, we found an additional 13 variants that did not achieve MAF > 0.01 but were observed once in homozygosity in the absence of the Fanconi anemia phenotype and are thus listed as “BS2-only variants” (Additional file 9: Table S9).

### Pathogenicity prediction and loss of function in reclassified variants

In Table 2, we summarize the number of pathogenic variants for each set of reclassified variants. For each variant in the supplementary tables, we report the pathogenicity predictions that were computed using SIFT, PolyPhen2, MutationTaster, MetaSVM, and CADD. We note that there are many reclassified variants that are predicted damaging, which underscores the cautionary note made by the ACMG guidelines with respect to how in silico prediction modules should be incorporated in weighing the evidence for pathogenicity. Similarly, the prevalence of reclassified variants that appear to be loss-of-function (LOF) (ranging from 1.4% in Criteria 4 for ClinVar to 18% in Criteria 1 in HGMD) is a reminder that an apparently LOF in a known disease gene is not a sufficient evidence to prove pathogenicity, which is also highlighted by the ACMG guidelines.

**Table 2:** Summary of pathogenicity and LOF of reclassified variants

HGMD			
Reclassification criteria	Total reclassified	Pathogenicity prediction (SIFT, PolyPhen, MutationTaster, metaSVM, CADD)	LOF (stop-gain, frameshift, splicing)
Criteria1: > = 0.05 (Additional file 1: Table S1)	255	9, 24, 16, 5, 77	13, 7, 15 (13.3%)
Criteria 2: > = 0.05 in SGP (and < 0.05 in PublicDB) (Additional file 3: Table S3)	16	5, 3, 8, 5, 12	2, 0, 1 (18%)
Criteria 3: > = 0.01 in SGP (at least 1 hom) (Additional file 5: Table S5)	607	97, 127, 149, 66, 288	34, 0, 16 (8.2%)
Criteria 4: <0.01, hom in SGP, no association with disease (Additional file 7: Table S7)	215	58, 84, 105, 60, 141	7, 0, 5 (5.5%)
	Total = 1093		
ClinVar			
Reclassification criteria	Total reclassified	Pathogenicity prediction (SIFT, PolyPhen, MutationTaster, metaSVM, CADD)	LOF (stop-gain, frameshift, splicing)
Criteria1 > = 0.05 (Additional file 2: Table S2)	334	23, 35, 11, 9, 65	2, 1, 6 (2.7%)
Criteria 2: > = 0.05 in SGP (and < 0.05 in PublicDB) (Additional file 4: Table S4)	10	1, 2, 4, 1, 6	0, 0, 1 (10%)
Criteria 3: > = 0.01 in SGP (at least 1 hom) (Additional file 6: Table S6)	484	69, 86, 70, 41, 165	3, 0, 5 (1.65%)
Criteria 4: <0.01, hom in SGP, no association with disease (Additional file 8: Table S8)	70	17, 25, 27, 21, 41	1, 0, 0 (1.4%)
	Total = 898		

For each set of reclassified variants, a summary of the pathogenicity as predicted by different software tools including, SIFT, Polyphen2, MutationTaster, MetaSVM, and CADD is given. Also a summary of LOF variants, including those with stop-gain, frameshifts, and splicing, is also given

## Discussion

The growing application of clinical genomics in the various disciplines of medicine has made the need for improved specificity of the medical annotation of the human genome a more acute problem than ever before. For example, even in a healthy individual free of Mendelian diseases, it is inevitable that clinical genomics will uncover the carrier status of at least one or more “pathogenic” variants [16]. Unfortunately, despite the increasing sophistication of bioinformatics tools, their ability to annotate variants as pathogenic or benign remains limited. This is clearly reflected by the ACMG guidelines that rightly place emphasis on empirical evidence rather than in silico prediction tools, especially the behavior of the variant in the human genetic pool, i.e. its segregation with the phenotype and population frequency. As more genomes are analyzed, the odds of encountering ultra-rare variants increase and these can be very challenging to classify given the very limited available data on these variants in the human population.

We have been exploiting the unique characteristics of the Saudi population to identify many pathogenic alleles in known and novel disease genes, thus contributing to the morbid genome of Mendelian diseases in humans [17, 18]. However, we show in this study that our population can also improve the specificity of the morbid genome map of human Mendelian diseases [19]. Not only do we demonstrate that many variants in question represent Arab-enriched variants, but we also take advantage of autozygosity to show that their presence in homozygosity does not lead to the reported phenotype, thus establishing two key lines of evidence in support of their likely benign nature. Although the latter, i.e. homozygosity without a phenotype, is not in itself sufficient to classify a variant as benign according to the ACMG guidelines, it does raise legitimate concerns about its claimed pathogenicity. By providing a full list of these variants as “BS2-only variants,” we hope the wider genetics community will find this list helpful as they weigh the evidence in the context in which they are encountered. Although every effort has been made to exclude the reported disease phenotype in these homozygotes, we note that issues related to non-penetrance, and atypical or mild phenotypes, cannot be fully ruled out. Furthermore, variants that are only expressed phenotypically when in trans with more severe variants will evade detection by our method, although we note that these tend to be rare.

Germline predisposition to cancer represents a peculiar class of Mendelian phenotypes. These typically dominant mutations have age-dependent penetrance, which forms the basis for their use in the primary prevention of these cancers when coupled with appropriate medical/surgical intervention [20]. On the other hand, this feature poses a major interpretation challenge since their presence in the “controls” cannot be taken as evidence against their pathogenicity. This is especially challenging in the case of such a common cancer as breast cancer, which affects up to 12% of the population (www.naaccr.org). Fortunately, the biallelic presence of pathogenic *BRCA2* alleles is associated with a specific fully penetrant clinical phenotype known as Fanconi anemia and may present as microcephalic primordial dwarfism in severe cases, whereas biallelic *BRCA1* mutation is presumably lethal in humans [21, 22]. Therefore, we hypothesized that we can challenge at least some of the previously reported *BRCA1* and *BRCA2* alleles by demonstrating their presence in homozygosity in individuals who lack the abovementioned phenotype. Indeed, we show that at least 18 of previously reported VOUS in BRCA1 and BRCA2 can be questioned as they have also been observed in our database in homozygosity. In addition, we confirm variants with pathogenic status in BRCA1 and BRCA2 as they were never encountered in homozygosity except in two cases with resulting Fanconi anemia phenotype (NM_ 000059.3:c.7007G > A:p.Arg2336His and NM_000059.3: c.9152delC, p.Pro3051Hisfs*11). In view of the highly invasive interventions recommended in patients with pathogenic BRCA1 or BRCA2 variants, VOUS in these genes are particularly challenging and we hope our efforts will help alleviate the anxiety and confusion associated with at least some of these variants.

## Conclusions

We show that the current version of the morbid genome suffers from specificity limitations and that available large sequencing initiatives in outbred populations do not fully address this challenge. The data we present from the SHGP database provide an example of the value that population-specific databases add to the ultimate goal of constructing a complete and highly specific map of the human morbid genome.

## Methods

### Human participants

All sequenced individuals have signed an informed consent form relevant to the disease with which they presented as per KFSHRC IRB-approved research protocols. These individuals were phenotyped for their suspected Mendelian diseases. Venous blood was collected for DNA extraction.

### The Saudi Human Genome Program database

The SHGP database is generated on individuals with various genetic diseases based on the Mendeliome assay and/or exome sequencing. As described before, the Mendeliome assay comprises 13 gene panels which cover the spectrum of “pediatric and adult” clinical genetic medicine [15]. Within each panel, genes were sorted based on the most prominent sign/symptom with which they are most likely to be associated upon presentation to clinical care. A total of 3070 genes covering over 4000 Mendelian disorders as annotated by OMIM up to August 2013 were used as a basis for the design and synthesis of highly multiplexed gene panels using Ion AmpliSeq Designer software (Life Technologies, Carlsbad, CA, USA). For both the Mendeliome assay as well as for exome sequencing, DNA samples were treated to obtain Ion Proton AmpliSeq libraries as appropriate. The template-positive Ion PI Ion Sphere particles were processed for sequencing on the Ion Proton instrument (Thermo Fisher, Carlsbad, CA, USA).

After running several quality checks as described before, the reads were aligned using the tmap program (Ion Torrent Suite, Thermo Fisher, Carlsbad, CA, USA, https://github.com/iontorrent/TS) to the reference hg19 sequence. The variants within the aligned reads were called using the Torrent Suite Variant Caller (TVC) program.

A total of 5849 non-overlapping individuals were assayed using the Mendeliome assay and 2000 were assayed using whole-exome sequencing as of August 2016. These comprise the content of the SHGP database until that data.

In addition to these, we sequenced additional 350 exomes from the sample pool using Illumina. That is, we have 350 samples whose exomes were sequenced using both the Ion Proton and Illumina platforms. The Illumina exomes were sequenced as follows: the exome target regions were captured using the TruSeq Exome Enrichment kit (Illumina) according to the recommended manufacturer’s protocol. Then, the individual samples were processed to produce Illumina sequencing libraries and, in the subsequent step, the sequencing libraries were enriched for the desired target using the Illumina Exome Enrichment protocol. The final libraries were then sequenced using an Illumina HiSeq 2500 Sequencer to an average read depth of target regions of 81.8X. The reads were mapped against UCSC hg19 by BWA. The SNVs and Indels were called using the GATK package.

For both Ion and Illumina exomes, the variants were annotated using public knowledge databases as well as in-house variants databases, as described in [15]. The pathogenicity predictions were computed using SIFT, PolyPhen2, MutationTaster, MetaSVM, and CADD.

### Variant quality

For this study, we used strict quality criteria for including the variants to assure correctness of conclusions. Variants were classified based on the SHGP database information only if:

1. they are called by both Proton and Illumina platforms; or
2. they are called by Ion Proton only but met all the following criteria: SNP (i.e. not indels) with a minimum quality score of 1000, depth of 150, and confirmed by Sanger.

About 99% of the SHGP database variants (in Additional file 3: Table S3; Additional file 4: Table S4; Additional file 5: Table S5; Additional file 6: Table S6; Additional file 7: Table S7; Additional file 8: Table S8; Additional file 9: Table S9) we report here and contribute to the reclassification outcome are confirmed by both platforms. In each of the Additional file 3: Table S3; Additional file 4: Table S4; Additional file 5: Table S5; Additional file 6: Table S6; Additional file 7: Table S7; Additional file 8: Table S8; Additional file 9: Table S9, we report the average quality score and depth for both Ion and Illumina platforms.

### Critical examination of the morbid genome of Mendelian diseases

We first queried HGMD for all variants with the designation “disease mutation” (DM), “?disease mutation” (DM?), “disease-associated polymorphism” (DP), and “disease-associated polymorphism with supporting functional evidence” (DFP). We also queried ClinVar for all variants with the designation “VOUS, pathogenic, or likely pathogenic”. We then examined the frequency of these variants according to 1000 Genomes, Kaviar, and ExAC. According to the ACMG guidelines, a population frequency of > 5% is sufficient to classify a variant as benign [14]. A frequency of < 5% but too high for the actual disease frequency is insufficient to confidently classify a variant as benign unless it can also be shown to be present in an individual who lacks the phenotype [14]. Since ExAC, Kaviar, and 1000 Genomes are mostly based on healthy individuals, we classified as benign dominant alleles with MAF > 0.01 and recessive alleles with MAF > 0.01 and present homozygous at least once. We then used our SHGP database to query the same list of variants and compared the results. To sum up, we used four criteria to reclassify the variants in HGMD and ClinVar:

- Criteria 1: reclassification based on allele frequency larger than or equal to 5% in public databases (1000 Genomes, ExAc, Kaviar).
- Criteria 2: reclassification based on allele frequency larger than or equal to 5% in the SHGP database and less than 5% in public databases.
- Criteria 3: reclassification based on allele frequency larger than or equal to 1% and exists in at least one homozygous state in the SHGP database in an individual who lacks the reported phenotype.
- Criteria 4: reclassification based on allele frequency less than 1% and exists at least once in homozygous state in the SHGP database in an individual who lacks the reported phenotype.

Because the SHGP database is heavily enriched for individuals with homozygous pathogenic mutations, we used the following formula to derive allele frequency without being biased by the number of diseased homozygotes: $SG{P}_{AF}=\frac{n{o}_{het}}{2\left({no}_{screened}-{no}_{hom}\right)}$

Additionally, we downloaded the entire list of *BRCA1* and *BRCA2* variants in the BRCA Exchange database (part of the Global Alliance) that are listed as pathogenic, likely pathogenic, or VOUS and analyzed them following the same procedure and criteria described above.

## Additional files

Additional file 1: Table S1. Reclassified HGMD variants based on high MAF in public databases. (PDF 174 kb)
Additional file 2: Table S2. Reclassified ClinVar variants based on high MAF in public databases. (PDF 194 kb)
Additional file 3: Table S3. Reclassified HGMD variants based on high MAF in the SHGP database despite low MAF in public databases. (PDF 130 kb)
Additional file 4: Table S4. Reclassified ClinVar variants based on high MAF in the SHGP database despite low MAF in public databases. (PDF 127 kb)
Additional file 5: Table S5. Reclassified HGMD variants based on MAF of 0.01 threshold and lack of phenotype in homozygotes. (PDF 273 kb)
Additional file 6: Table S6. Reclassified ClinVar variants based on MAF of 0.01 threshold and lack of phenotype in homozygotes. (PDF 285 kb)
Additional file 7: Table S7. Reclassified HGMD variants based on lack of phenotype in homozygotes BS2-only variants. (PDF 184 kb)
Additional file 8: Table S8. Reclassified ClinVar variants based on lack of phenotype in homozygotes BS2-only variants. (PDF 154 kb)
Additional file 9: Table S9. Reclassified *BRCA* variants based on lack of phenotype in homozygotes. (PDF 125 kb)
