# Supplementary material for: Revisiting the morbid genome of Mendelian disorders
Source: Genome Biol. 2016 Nov 24;17:235. doi: 10.1186/s13059-016-1102-1 (PMC5123336; doi:10.1186/s13059-016-1102-1)
Supplement: Additional file 1: Table S1. — Reclassified HGMD variants based on high MAF in public databases. (PDF 174 kb) [file 13059_2016_1102_MOESM1_ESM.pdf]

**Table S1: Reclassified HGMD variants based on high MAF in public databases**

Column titled 1000G includes frequencies in 1000G, columns titled "KAVIAR_AF, KAVIAR_AC" include allele frequencies and counts in KAVIAR DB, column titled "EXAC_ALL" includes average EXAC fquency over different populations, and subsequent EXAC columns include frequencies for each popoulation, column titled "HGMD2016" includes HGMD record, column titled "SGP_screene" includes number of screenees for that variant from gene panels and exomes, "SGP_C" includes number of occurrences of the vatiant, "SGP_C_hom" includes occurrences as hom, columns titled "SGP_Freq" and "SGP_Freq_hom" include frequency for all and homozygous ones, "SGP_AF_Strict" includes computation of allele frequency using formula given in the paper, columns titled "SIFT", "PolyPhen", "MutationTaster", "MetaSVM", and "cadd phred" include pahogeneicity predictions using respective tools.

| chromosom | start pos | end pos | reference | observed | refGene ge | refGene fur | refGene sp | refGene exonic functio | refGene AA | 1000G all | Kaviar_AF | Kaviar_AC | Kaviar_AN | ExAC_ALL | ExAC_AFR | ExAC_AMR | ExAC_EAS | ExAC_FIN | ExAC_NFE | ExAC_OTH | ExAC_SAS | HGMD2016 | SGP_Scree | SGP_C | SGP_C_hor | SGPFreq | SGPFreqHc | SGP_AF_Stric | SIFT | PolyPhen 2 | LJB Mutatic | MetaSVM_s | CADD_phred |
|---|---|---|---|---|---|---|---|---|---|---|---|---|---|---|---|---|---|---|---|---|---|---|---|---|---|---|---|---|---|---|---|---|---|
| 1 | 16383682 | 16383682 | G | C | CLCNKB | UTR3 | NM_000085:c.*271G>C,NM_001165945:c.*271G>C | | | 0 | 0.0109895 | 1699 | 154602 | 0.3197 | 0.399 | 0.2611 | 0.4272 | 0.4153 | 0.3183 | 0.3533 | 0.2964 | Info=CLASS=DM?;MUT=REF;ID=CR133355;Ref=G;Alt= | 3753 | 183 | 2 | 0.04876099 | 0.00053291 | 0.024120469 | . | . | N | T | 0.058 |
| 3 | 11314041 | 11314041 | A | G | ATG7 | UTR5 | NM_001136031:c.-26129A>G,NM_006395:c.-261 | | | 0.919129 | 0.0002689 | 7 | 26028 | | | | | | | | | Info=CLASS=DM?;MUT=REF;ID=CR130996;Ref=A;Alt= | 3219 | 359 | 287 | 0.11152532 | 0.08915812 | 0.011705414 | | | | | |
| 1 | 115576023 | 115576023 | A | G | TSHB | exonic | | nonsynonymous SNV | TSHB:NM_( | 0.983626 | 0.952226 | 147216 | 154602 | 0.9707 | 0.9955 | 0.9929 | 1 | 0.944 | 0.9645 | 0.9692 | 0.9599 | Info=CLASS=DM?;MUT=REF;ID=CM991218;Ref=A;Alt= | 2492 | 2416 | 2342 | 0.96950241 | 0.93980738 | 0.028009084 | 0.761 | B | P | T | 0.001 |
| 1 | 100672060 | 100672060 | T | C | DBT | exonic | | nonsynonymous SNV | DBT:NM_00 | 0.891773 | 0.870765 | 134622 | 154602 | 0.9138 | 0.7642 | 0.9628 | 0.9703 | 0.9338 | 0.9096 | 0.9306 | 0.9521 | Info=CLASS=DM?;MUT=REF;ID=CM983430;Ref=T;Alt= | 3411 | 2757 | 2240 | 0.80826737 | 0.65669892 | 0.112832824 | 1 | B | P | T | 1.112 |
| 1 | 98348885 | 98348885 | G | A | DPYD | exonic | | nonsynonymous SNV;r | DPYD:NM_( | 0.739816 | 0.757623 | 117130 | 154602 | 0.7652 | 0.5836 | 0.7928 | 0.9258 | 0.7035 | 0.7788 | 0.7737 | 0.7456 | Info=CLASS=DM?;MUT=REF;ID=CM970421;Ref=G;Alt= | 4580 | 3203 | 2190 | 0.69934498 | 0.47816594 | 0.145337159 | | | | | |
| 15 | 58853079 | 58853079 | C | A | LIPC;LIPC | exonic | | nonsynonymous SNV;r | LIPC:NM_0( | 0.936102 | 0.951204 | 147058 | 154602 | 0.9687 | 0.8572 | 0.9902 | 0.9375 | 0.9476 | 0.9838 | 0.9801 | 0.9875 | Info=CLASS=DM?;MUT=REF;ID=CM962679;Ref=C;Alt= | 3332 | 2763 | 2670 | 0.82923169 | 0.80132053 | 0.023284927 | 0.495 | B | P | T | 22.8 |
| 13 | 32929387 | 32929387 | T | C | BRCA2 | exonic | | nonsynonymous SNV;r | BRCA2:NM_ | 0.975839 | 0.976068 | 150902 | 154602 | 0.9937 | 0.933 | 0.997 | 0.9999 | 1 | 0.9996 | 0.9989 | 0.9998 | Info=CLASS=DM?;MUT=REF;ID=CM960194;Ref=T;Alt= | 3420 | 2833 | 2767 | 0.82836257 | 0.80906433 | 0.016204272 | 1 | . | P | T | 0.372 |
| 5 | 73981270 | 73981270 | T | C | HEXB | exonic | | nonsynonymous SNV | HEXB:NM_( | 0.98143 | 0.0048059 | 743 | 154602 | 0.9708 | 0.9923 | 0.9834 | 0.9999 | 0.9789 | 0.962 | 0.9725 | 0.9659 | Info=CLASS=DM?;MUT=REF;ID=CM952225;Ref=T;Alt= | 3222 | 2423 | 2361 | 0.75201738 | 0.73277467 | 0.015184913 | 1 | . | P | T | 0.1 |
| 14 | 88401213 | 88401213 | T | C | GALC;GALC | exonic | | nonsynonymous SNV;r | GALC:NM_( | 0.940695 | 0.961411 | 148636 | 154602 | 0.9828 | 0.8607 | 0.9918 | 0.9375 | 1 | 0.9996 | 0.9922 | 0.9975 | Info=CLASS=DM?;MUT=REF;ID=CM950521;Ref=T;Alt= | 4580 | 3360 | 3252 | 0.73362445 | 0.71004367 | 0.018280298 | 1 | B | P | T | 0.001 |
| X | 31496350 | 31496350 | C | T | DMD | exonic | | nonsynonymous SNV | DMD:NM_0 | 0.881854 | 0.0006662 | 103 | 154602 | 0.9045 | 0.9888 | 0.7877 | 0.9234 | 0.9421 | 0.9459 | 0.93 | 0.7103 | Info=CLASS=DM?;MUT=REF;ID=CM135935;Ref=C;Alt= | 8171 | 4960 | 4742 | 0.60702484 | 0.58034512 | 0.018793103 | 0.624 | B | P | T | 17.1 |
| 19 | 44047826 | 44047826 | T | A | XRCC1;XRC | exonic | | nonsynonymous SNV | XRCC1:NM_ | 1 | 0.0063 | 974 | 154602 | 1 | 0.9999 | 0.9999 | 1 | 1 | 1 | 1 | 1 | Info=CLASS=DM?;MUT=REF;ID=CM1313960;Ref=T;Alt= | 3206 | 2783 | 2606 | 0.86805989 | 0.8128509 | 0.046505518 | | | | | |
| 7 | 143017807 | 143017807 | G | T | CLCN1 | exonic | | nonsynonymous SNV | CLCN1:NM_ | 0.99361 | 0.0001035 | 16 | 154602 | 0.9861 | 0.9959 | 0.9959 | 1 | 0.9828 | 0.9783 | 0.9879 | 0.9982 | Info=CLASS=DM?;MUT=REF;ID=CM127929;Ref=G;Alt= | 4769 | 3533 | 3439 | 0.74082617 | 0.72111554 | 0.015412363 | 1 | B | P | T | 18.83 |
| 15 | 45392075 | 45392075 | G | A | DUOX2;DU( | exonic | | nonsynonymous SNV;r | DUOX2:NM | 0.720248 | 0.8492 | 131288 | 154602 | 0.8644 | 0.2553 | 0.8539 | 0.9425 | 0.9273 | 0.9248 | 0.9049 | 0.942 | Info=CLASS=DM?;MUT=REF;ID=CM085371;Ref=G;Alt= | 2492 | 2260 | 1660 | 0.90690209 | 0.66613162 | 0.180505415 | 0.043 | B | P | T | 23 |
| 1 | 169521849 | 169521849 | T | C | F5 | exonic | | synonymous SNV | F5:NM_000 | 0.0830671 | 0.0844038 | 13049 | 154602 | 0.0862 | 0.0874 | 0.0997 | 0.0627 | 0.0561 | 0.0859 | 0.0727 | 0.1028 | Info=CLASS=DM?;MUT=REF;ID=CM070088;Ref=T;Alt= | 4580 | 382 | 24 | 0.08340611 | 0.00524017 | 0.039185639 | | | | | |
| 3 | 33055721 | 33055721 | A | G | GLB1 | exonic | | nonsynonymous SNV;r | GLB1:NM_( | 0.927117 | 0.941262 | 145521 | 154602 | 0.98 | 0.7803 | 0.9877 | 1 | 1 | 0.9986 | 0.9878 | 0.9995 | Info=CLASS=DM?;MUT=REF;ID=CM051080;Ref=A;Alt= | 7120 | 4627 | 4455 | 0.64985955 | 0.62570225 | 0.017577925 | 0.915 | B | P | T | 6.854 |
| 16 | 56904587 | 56904587 | C | G | SLC12A3 | exonic | | nonsynonymous SNV;r | SLC12A3:N | 0.990615 | 0.96557 | 149279 | 154602 | 0.9756 | 0.9929 | 0.9946 | 0.9997 | 0.9631 | 0.9635 | 0.9934 | 0.9916 | Info=CLASS=DM?;MUT=REF;ID=CM022671;Ref=C;Alt= | 5338 | 3737 | 3695 | 0.70007493 | 0.69220682 | 0.00601633 | 1 | B | P | T | 8.224 |
| 8 | 145640411 | 145640411 | A | G | SLC39A4 | exonic | | nonsynonymous SNV | SLC39A4:N | 0.984625 | 0.953804 | 147460 | 154602 | 0.9678 | 0.9933 | 0.9868 | 0.9997 | 0.9482 | 0.9536 | 0.9754 | 0.9862 | Info=CLASS=DM?;MUT=REF;ID=CM021685;Ref=A;Alt= | 5731 | 2344 | 2334 | 0.40900366 | 0.40725877 | 0.00109553 | | | | | |
| 5 | 149360877 | 149360877 | T | C | SLC26A2 | exonic | | nonsynonymous SNV | SLC26A2:N | 0 | 0.94132 | 145530 | 154602 | 0.9928 | 0.9983 | 0.9977 | 1 | 0.9982 | 0.9891 | 0.9956 | 0.9947 | Info=CLASS=DM?;MUT=REF;ID=CM016008;Ref=T;Alt= | 3316 | 2821 | 2805 | 0.85072376 | 0.84589867 | 0.00418082 | 1 | B | P | T | 0.011 |
| 12 | 122295335 | 122295335 | T | C | HPD;HPD | exonic | | nonsynonymous SNV;r | HPD:NM_0( | 0.876398 | 0.834562 | 129025 | 154602 | 0.8502 | 0.9622 | 0.7197 | 0.852 | 0.775 | 0.8635 | 0.8524 | 0.8465 | Info=CLASS=DM?;MUT=REF;ID=CM003889;Ref=T;Alt= | 3222 | 2740 | 2351 | 0.85040348 | 0.72967101 | 0.095040313 | 1 | . | P | T | 1.004 |
| 11 | 88045583 | 88045583 | A | G | CTSC | exonic | | nonsynonymous SNV;r | CTSC:NM_( | 0.930911 | 0.874187 | 135151 | 154602 | 0.8861 | 0.9139 | 0.923 | 0.9995 | 0.8931 | 0.846 | 0.8767 | 0.9427 | Info=CLASS=DM?;MUT=REF;ID=CM002936;Ref=A;Alt= | 2767 | 2626 | 1971 | 0.94904228 | 0.71232382 | 0.183833848 | 0.734 | B | P | T | 0.003 |
| 7 | 6026775 | 6026775 | T | C | PMS2 | exonic | | nonsynonymous SNV;r | PMS2:NM_( | 0.883187 | 0.831263 | 128515 | 154602 | 0.8514 | 0.9229 | 0.6563 | 0.9238 | 0.8432 | 0.855 | 0.8381 | 0.8961 | Info=CLASS=DM?;MUT=REF;ID=BM1497492;Ref=T;Alt= | 2981 | 2698 | 2046 | 0.90506541 | 0.68634686 | 0.166496425 | 1 | B | P | T | 0.135 |
| 8 | 143956693 | 143956693 | G | A | CYP11B1 | exonic | | nonsynonymous SNV | CYP11B1:N | 0 | 0.0669396 | 10349 | 154602 | 0.0739 | 0.0462 | 0.1282 | 0.3965 | 0.0284 | 0.0185 | 0.0803 | 0.1399 | Info=CLASS=DM?;MUT=ALT;ID=HM972177;Ref=G;Alt= | 3866 | 278 | 18 | 0.07190895 | 0.00465598 | 0.033704952 | 1 | B | P | T | 0.034 |
| 6 | 134210600 | 134210600 | G | T | TCF21 | exonic | | nonsynonymous SNV;r | TCF21:NM_ | 0.0595048 | 0.0166233 | 2570 | 154602 | 0.0172 | 0.1778 | 0.0092 | 0.002 | 0 | 0.0011 | 0.0143 | 0.0017 | Info=CLASS=DM?;MUT=ALT;ID=HM050002;Ref=G;Alt= | 3753 | 93 | 4 | 0.02478018 | 0.00106581 | 0.011863503 | 0.027 | B | P | T | 14.3 |
