# Supplementary material for: Revisiting the morbid genome of Mendelian disorders
Source: Genome Biol. 2016 Nov 24;17:235. doi: 10.1186/s13059-016-1102-1 (PMC5123336; doi:10.1186/s13059-016-1102-1)
Supplement: Additional file 2: Table S2. — Reclassified ClinVar variants based on high MAF in public databases. (PDF 194 kb) [file 13059_2016_1102_MOESM2_ESM.pdf]

Table S2. Reclassified ClinVar variants based on high MAF in public databases

Column titled 1000G includes frequencies in 1000G, columns titled "KAVIAR_AF, KAVIAR_AC" include allele frequencies and counts in KAVIAR DB, column titled "EXAC_ALL" includes average EXAC frquency ove populations, and subsequent EXAC columns include frequencies for each popoulation, columns titled "CLNSDB" to "CLNSIG" includes ClinVar records where "CLINSIG" includes variant class in ClinVar, column titled "SGP_screene" includes number of screenees for that variant from gene panels and exomes, "SGP_C" includes number of occurences of the vatiant, "SGP_C_hom" includes occurences as hom, columns titled "SGP_Freq" and "SGP_Freq_hom" include frequency for all and homozygous ones, "SGP_AF_Strict" includes computation of allele frequency using formula given in the paper, columns titled "SIFT", "PolyPhen", "MutationTaster", "MetaSVM", and "cadd phred" include pahogeneicity predictions using respective tools.

chromosome	start pos	end pos	reference	observed	refGene gene	refGene function	refGene splice info	refGene exonic fun	refGene AA change	1000G all	Kaviar_AF	Kaviar_AC	Kaviar_AN	CLNDSDB	CLNACC	CLNDBN	CLNDSDBID	CLINSIG
1	98348885	98348885	G	A	DPYD	exonic		nonsynonymous SN	DPYD:NM_000110:	0.739816	0.757623	117130	154602	MedGen:OMIM\x2c	RCV000000464.2\x	Dihydropyrimidine_d	C2720286:274270\x	Pathogenic\x2cnot p
1	100672060	100672060	T	C	DBT	exonic		nonsynonymous SN	DBT:NM_001918:ex	0.891773	0.870765	134622	154602	MedGen\x2cMedGen	RCV000012727.23\	Intermediate_maple_	CN069615\x2cCN16	Pathogenic\x2cother
1	115236057	115236057	G	A	AMPD1;AMPD1	exonic		stopgain	AMPD1:NM_000036	0.038139	0.0005498	85	154602	MedGen:OMIM:SNC	RCV000019933.29	Muscle_AMP_deam	C0268123:615511:9	Pathogenic
1	159174683	159174683	T	C	ACKR1	UTR5	NM_002036:c.-67T>C			0.266374	0.0139714	2160	154602	.|.|MedGen:OMIM	RCV000000006.4|R	DUFFY_BLOOD_GF	.|.|C2676078:611862	Pathogenic|other|other
1	161518333	161518333	A	T	FCGR3A	exonic		nonsynonymous SN	FCGR3A:NM_0005	0.0265575	0.0459308	7101	154602	MedGen:OMIM	RCV000015953.3	Immunodeficiency_20	C3810342:615707	Pathogenic
1	161599571	161599571	T	C	FCGR3B;FCGR3B	exonic		nonsynonymous SN	FCGR3B:NM_00124	0	0.272635	42150	154602	.	RCV000030607.2	Neutrophil-specific_a	.	Pathogenic
1	161599643	161599643	T	C	FCGR3B	exonic		nonsynonymous SN	FCGR3B:NM_00124	0.283546	0.124248	19209	154602	.	RCV000030607.2	Neutrophil-specific_a	.	Pathogenic
1	161599693	161599693	T	C	FCGR3B	exonic		nonsynonymous SN	FCGR3B:NM_00124	0.532947	0.0381237	5894	154602	.	RCV000030607.2	Neutrophil-specific_a	.	Pathogenic
1	161599779	161599779	G	C	FCGR3B	exonic		nonsynonymous SN	FCGR3B:NM_00124	0.127596	0.0139325	2154	154602	.	RCV000030607.2	Neutrophil-specific_a	.	Pathogenic
1	169519049	169519049	T	C	F5;F5	exonic		nonsynonymous SN	F5:NM_000130:exon	0.99401	0.967749	149616	154602	MedGen|.|MedGen	RCV000000674.2|R	Thrombophilia_due_	C2674152|.|C0000	Pathogenic|other|oth
1	171076966	171076966	G	A	FMO3;FMO3	exonic		nonsynonymous SN	FMO3:NM_0010022	0.347843	0.379762	58712	154602	.|MedGen:OMIM:SN	RCV000017711.5|R	Trimethylaminuria\x2	.|C0342739:602079:	Pathogenic|Likely pa
1	171080080	171080080	G	A	FMO3	exonic		nonsynonymous SN	FMO3:NM_0010022	0.0976438	0.0796626	12316	154602	MedGen:OMIM:SNC	RCV000017698.27	Trimethylaminuria	C0342739:602079:2	Pathogenic
1	171083242	171083242	A	G	FMO3	exonic		nonsynonymous SN	FMO3:NM_0010022	0.0914537	0.153963	23803	154602	.|MedGen:OMIM:SN	RCV000017711.5|R	Trimethylaminuria\x2	.|C0342739:602079:	Pathogenic|Likely pa
1	196659237	196659237	C	T	CFH	exonic		nonsynonymous SN	CFH:NM_000186:ex	0.733427	0.0001229	19	154602	MedGen:OMIM|Med	RCV000018015.4|R	Age-related_macula	C1853147:610698|C	other|Pathogenic
1	209961970	209961970	C	G	IRF6;IRF6	exonic		nonsynonymous SN	IRF6:NM_00120669	0	0.0000323	5	154602	MedGen:OMIM:ORF	RCV000087748.4	Van_der_Woude_sy	C0175697:119300:8	Pathogenic
1	231408091	231408091	A	G	GNPAT	exonic		nonsynonymous SN	GNPAT:NM_001316	0.135583	0.160334	24788	154602	MedGen:OMIM:ORF	RCV000029140.3	Rhizomelic_chondro	C1857242:222765:3	Pathogenic
2	44066247	44066247	G	C	ABCG8;ABCG8	exonic		nonsynonymous SN	ABCG8:NM_022437	0.0605032	0.0207501	3208	154602	MedGen:OMIM	RCV000005263.3	Gallbladder_disease	C1969115:611465	Pathogenic
2	48025764	48025764	C	T	MSH6;MSH6	exonic		synonymous SNV	MSH6:NM_0012814	0.0285543	0.0735437	11370	154602	MedGen:Orphanet:S	RCV000075015.2\x	Lynch_syndrome\x2c	C1333990:ORPHA1	Pathogenic\x2cPath
2	63131731	63131731	G	A	EHBP1	intronic				0.0944489	0.155333	4043	26028	MedGen:OMIM	RCV000001429.2	Prostate_cancer\x2c	C2678479:611868	Pathogenic
2	190925077	190925077	T	C	MSTN	exonic		nonsynonymous SN	MSTN:NM_005259:	0.0710863	0.0303166	4687	154602	MedGen:OMIM	RCV000055914.1	Muscle_hypertrophy	C0236033:614160	Pathogenic
2	209010778	209010778	C	-	LOC100507443	ncRNA_intronic				0.134984	0.0039262	607	154602	MedGen:OMIM	RCV000034855.33	Cataract_39\x2c_mu	C3808800:615188	Pathogenic
2	233243586	233243586	C	T	ALPP;ALPP	exonic		nonsynonymous SN	ALPP:NM_001632:e	0.0844649	0.0799925	12367	154602	.|.\x2c.|	RCV000014644.2|R	ALKALINE_PHOSPI	.|.\x2c.|	Benign|Pathogenic\x
2	233390199	233390199	C	G	PRSS56	exonic		nonsynonymous SN	PRSS56:NM_00119	0.0213658	0.0123543	1910	154602	MedGen:OMIM	RCV000024374.2	Microphthalmia\x2c_	C3150757:613517	Pathogenic
[illegible]																		

refGene gene	ExAC_ALL	ExAC_AFR	ExAC_AMR	ExAC_EAS	ExAC_FIN	ExAC_NFE	ExAC_OTH	ExAC_SAS	SGP_Screenee	SGP_C	SGP_C_hom	SGPFreq	SGPFreqHom	SGP_AF_Strict	SIFT	PolyPhen 2 HDIV p	LJB MutationTaste	MetaSVM_pred	CADD_phred
DPYD	0.7652	0.5836	0.7928	0.9258	0.7035	0.7788	0.7737	0.7456	4580	3203	2190	0.699344978	0.478165939	0.145337159					
DBT	0.9138	0.7642	0.9628	0.9703	0.9338	0.9096	0.9306	0.9521	3411	2757	2240	0.80826737	0.656698915	0.112832824	1	B	P	T	1.112
AMPD1;AMPD1	0.0871	0.0205	0.0405	0	0.1181	0.1264	0.0929	0.0356	5417	338	19	0.06239616	0.003507476	0.02949607	.	.	P	.	36
ACKR1									2379	1393	675	0.585540143	0.283732661	0.17585109					
FCGR3A	0.0544	0.0111	0.0372	0	0.0643	0.0782	0.0661	0.0215	4786	230	4	0.048056832	0.000835771	0.023620401	0.122	D	N	T	0.466
FCGR3B;FCGR3B	0.3374	0.2413	0.4937	0.5589	0.33	0.2852	0.313	0.3962	4786	54	15	0.011282908	0.003134141	0.004080778	0.585	B	P	T	0.001
FCGR3B	0.0799	0.1058	0.1343	0.2264	0.0294	0.0493	0.0671	0.117	4786	11	9	0.00229837	0.001880485	0.000209139	0.172	B	N	T	0.002
FCGR3B	0.5887	0.5896	0.4302	0.3522	0.6137	0.6512	0.6191	0.5695	4786	2846	2639	0.594651066	0.551399916	0.029857205	1	.	P	T	0.001
FCGR3B	0.0001	0	0	0.0002	0	0	0	0.0008	4786	0	0	0	0	0	1	B	P	T	0.002
F5;F5	0.9785	0.9956	0.9939	1	0.9793	0.9687	0.9713	0.9853	4580	3441	3400	0.751310044	0.742358079	0.007118056					
FMO3;FMO3	0.383	0.4666	0.3075	0.2031	0.4513	0.413	0.4064	0.3273	3411	1297	268	0.380240399	0.078569335	0.157003357	0.562	P	P	T	21.6
FMO3	0.0805	0.0367	0.0869	0.2004	0.0461	0.0701	0.0672	0.0976	3411	702	81	0.205804749	0.023746702	0.09212283	0.075	B	P	T	4.624
FMO3	0.1529	0.0386	0.0848	0.1832	0.2492	0.1949	0.1435	0.0491	3411	237	7	0.069481091	0.002052184	0.033749083	0.199	D	P	T	23.9
CFH	0.6721	0.6307	0.8458	0.9506	0.5582	0.6169	0.6589	0.7008	6962	3973	2117	0.570669348	0.304079288	0.157194884	1	B	P	T	0.001
IRF6;IRF6	0.0711	0.1142	0.2001	0.165	0.2251	0.0518	0.0539	0.0273	3316	7	0	0.002110977	0	0.001055489	0.001	D	D	D	34
GNPAT	0.1612	0.0893	0.1093	0.1161	0.077	0.197	0.1652	0.1558	4156	1232	192	0.296438884	0.046198268	0.128078818	0.122	B	P	T	17.87
ABCG8;ABCG8	0.0582	0.0748	0.0498	0.0101	0.0882	0.0774	0.0323	0.0329	3720	442	42	0.118817204	0.011290323	0.054066667	0.045	P	N	T	22.7
MSH6;MSH6	0.0735	0.0176	0.0278	0	0.1564	0.1053	0.0621	0.017	2981	31	0	0.010399195	0	0.005199597					
EHBP1									2379	0	0	0	0	0					
MSTN	0.031	0.2075	0.0198	0.0026	0.0023	0.0175	0.031	0.0081	4577	369	20	0.080620494	0.004369674	0.03820889	0.667	B	P	T	9.742
LOC100507443	0.1398	0.1911	0.1011	0.089	0.1369	0.1419	0.1377	0.154	2379	373	21	0.156788567	0.008827238	0.074306634					
ALPP;ALPP	0.0819	0.04	0.0384	0.2248	0.062	0.0829	0.0861	0.0676	2379	171	12	0.071878941	0.005044136	0.033501896	0.027	D	P	T	23.7
PRSS56	0.105	0.0426	0.2632	0	0	0.1571	0.1087	0.0894	3990	159	14	0.03984924	0.003508772	0.01820236	.	.	.	.	4.696
[illegible]																			

	21	46957794	46957794	T	C	SLC19A1	exonic	nonsynonymous SN	SLC19A1:NM_0012	0.488618	0.511261	79042	154602	MedGen:OMIM:ORF	RCV000144916.1	Gastrointestinal_Strc	C0238198:606764:4	Uncertain significanc	0.577	0.4155	0.5903	0.5182	0.6026	0.5962	0.5727	0.6065	3219	1970	691	0.611991302	0.214682939	0.222550896	0.361	B	P	T	0.025
	21	47420726	47420726	C	G	COL6A1	intronic			0.830272	0.0002393	37	154602	MedGen\x2cMedGen	RCV000079769.4\x2	not_specified\x2cnot	CN169374\x2cCN22	Benign\x2cUncertain	0.8511	0.8015	0.929	0.8647	0.8834	0.8579	0.8433	0.7808	4577	2380	1393	0.519991261	0.304347826	0.127174333					
	21	47420726	47420726	C	T	COL6A1	intronic			0.0361422	0.822007	127084	154602	MedGen\x2cMedGen	RCV000079769.4\x2	not_specified\x2cnot	CN169374\x2cCN22	Benign\x2cUncertain	0.0107	0.1016	0.0091	0	0	0.0018	0.0132	0.0003	4577	172	30	0.0375792	0.006554512	0.015563349					
	22	18900750	18900750	G	A	PRODH	exonic	synonymous SNV	PRODH:NM_00119	0.763379	0.593783	91800	154602	MedGen	RCV000115064.1	not_provided	CN221809	Uncertain significanc	0.789	0.6471	0.7979	0.8412	0.8219	0.8084	0.7432	0.807	3222	2289	1161	0.710428305	0.360335196	0.213515048					
X		32669122	32669122	G	A	DMD	intronic			0.098543	0.0229753	598	26028	MedGen	RCV000080849.3	not_provided	CN221809	Uncertain significance									8171	86	33	0.010525028	0.004038673	0.003249739					
X		32669246	32669246	-	TCC	DMD	intronic			0.0988079	0.0043031	112	26028	MedGen	RCV000080850.3	not_provided	CN221809	Uncertain significance									8171	0	0	0	0	0					
X		100624842	100624842	-	T	BTK	intronic			0.10543	0.0121024	315	26028	MedGen:OMIM:ORF	RCV000029412.1	X-linked_agammagk	C0221026:300755:4	Uncertain significance									6337	0	0	0	0	0					
X		153640406	153640406	C	T	TAZ	splicing	NM_001303465:exon2:c.164-17C>T,NM_181313:exon2:c.110-		0.0649007	0.155768	24082	154602	MedGen:OMIM:ORF	RCV000030474.1	3-Methylglutaconic_a	C0574083:302060:1	Uncertain significanc	0.2006	0.0359	0.075	0.0002	0.3642	0.308	0.2013	0.0523	7192	420	170	0.05839822	0.023637375	0.017588293					
X		153792156	153792156	C	T	IKBKG	splicing	NM_001099856:exon9:c.1260-18C>T,NM_001099857:exon9:c		0	0.0000647	10	154602	MedGen:OMIM	RCV000030047.1	Hypohidrotic_ectode	C1846006:300291	Uncertain significanc	0.0541	0.0476	0.1667	0 .		0.0488 .	.		7094	1	1	0.000140964	0.000140964	0					
X		153792168	153792168	T	C	IKBKG	splicing	NM_001099856:exon9:c.1260-6T>C,NM_001099857:exon9:c.1		0	0.0076584	1184	154602	MedGen:OMIM	RCV000030048.1	Hypohidrotic_ectode	C1846006:300291	Uncertain significanc	0.3283	0.4576	0.25	0.5 .		0.2273	1	0.5	7094	11	9	0.001550606	0.001268678	0.000141054					
