# Supplementary material for: Revisiting the morbid genome of Mendelian disorders
Source: Genome Biol. 2016 Nov 24;17:235. doi: 10.1186/s13059-016-1102-1 (PMC5123336; doi:10.1186/s13059-016-1102-1)
Supplement: Additional file 5: Table S5. — Reclassified HGMD variants based on MAF of 0.01 threshold and lack of phenotype in homozygotes. (PDF 273 kb) [file 13059_2016_1102_MOESM5_ESM.pdf]

Table S5. Reclassified HGMD variants based on MAF of 0.01 threshold and lack of phenotype in homozygotes

Column titled 1000G includes frequencies in 1000G, columns titled "KAVIAR_AF, KAVIAR_AC" include allele frequencies and counts in KAVIAR DB, column titled "EXAC_ALL" includes average EXAC frquency over different populations, and subsequent EXAC columns include frequencies for each popoulation, column titled "HGMD2016" includes HGMD record, column titled "SGP_screene" includes number of screenees for that variant from gene panels and exomes, "SGP_C" includes number of occurences of the vatiant, "SGP_C_hom" includes occurences as hom, columns titled "SGP_Freq" and "SGP_Freq_hom" include frequency for all and homozygous ones, "SGP_AF_Strict" includes computation of allele frequency using formula given in the paper, columns titled "SIFT", "PolyPhen", "MutationTaster", "MetaSVM", and "cadd phred" include pahogenecity predictions using respective tools. Columns titled "AvgQIon"" and "AvgQIllum" include average quality scores for Ion and Illumina, and columns titled "AvgIonDepth" and "AvgIlluDepth" include average depth for Ion and Illumina respectively.

chromosome	start pos	end pos	reference	observed	AvgQION	AvgQIllum	AvgIonDepth	AvgIlluDepth	refGene gene	refGene function	refGene splice i	refGene exonic	refGene AA cha	HGMD2016	SGP_C	SGP_C_hom	SGP_Screenee	SGPFreq	SGPFreqHom	SGP_AF_Strict	1000G all	Kaviar_AF	Kaviar_AC	Kaviar_AN	ExAC_ALL	ExAC_AFR	ExAC_AMR	ExAC_EAS	ExAC_FIN	ExAC_NFE	ExAC_OTH	ExAC_SAS	SIFT	PolyPhen 2 HDI	LJB MutationTa	MetaSVM_pred	CADD_phred
1	16383682	16383682	G	C	1214.42	31.83877778	149	67.11111111	CLCNKB	UTR3	NM_000085:c.*271G>C,NM_001165945:c.*271G>C			Info=CLASS=DM?;MUT=F	182	2	3351	0.054312146	0.000596837	0.026865672	0	0.0109895	1699	154602	0.3197	0.399	0.2611	0.4272	0.4153	0.3183	0.3533	0.2964	.	.	N	T	0.058
1	115576023	115576023	A	G	1890.351445	75.96815009	399	13.91666667	TSHB	exonic		nonsynonymous	TSHB:NM_0005	Info=CLASS=DM?;MUT=F	1966	1908	2108	0.932637571	0.90512334	0.025129983	0.983626	0.952226	147216	154602	0.9707	0.9955	0.9929	1	0.944	0.9645	0.9692	0.9599	0.761	B	P	T	0.001
8	41552213	41552213	G	A	1442.3	31.36363636	237.6363636	14.09090909	ANK1	exonic		nonsynonymous	ANK1:NM_00003	Info=CLASS=DM?;MUT=F	11	1	2682	0.004101417	0.000372856	0.001864628	0.0153754	0.0042302	654	154602	0.0045	0.0497	0.0019	1	0	0.0000902	0.0033	0	0.305	B	D	T	11.36
1	100672060	100672060	T	C	515.7012195	64.93877005	229.3536585	12.09425287	DBT	exonic		nonsynonymous	DBT:NM_001918	Info=CLASS=DM?;MUT=F	2311	1882	3018	0.7657389	0.623591783	0.103273953	0.891773	0.870765	134622	154602	0.9138	0.7642	0.9628	0.9703	0.9338	0.9096	0.9306	0.9521	1	B	P	T	1.112
1	98348885	98348885	G	A	793.1814744	79.96033994	136.3680782	22.1388102	DPYD	exonic		nonsynonymous	DPYD:NM_0001	Info=CLASS=DM?;MUT=F	2646	1811	3920	0.675	0.461989796	0.138497263	0.739816	0.757623	117130	154602	0.7652	0.5836	0.7928	0.9258	0.7035	0.7788	0.7737	0.7456					
15	58853079	58853079	C	A	722.3266667	57.45225464	291.2222222	14.58333333	LIPC;LIPC	exonic;splicing		nonsynonymous	LIPC:NM_00023	Info=CLASS=DM?;MUT=F	2312	2234	2936	0.78746594	0.76089183	0.021440352	0.938102	0.951204	147058	154602	0.9687	0.8572	0.9902	0.9375	0.9476	0.9838	0.9801	0.9875	0.495	B	P	T	22.8
13	32929387	32929387	T	C	1083.962426	77.98181818	150.6363636	11.41818182	BRCA2	exonic		nonsynonymous	BRCA2:NM_000	Info=CLASS=DM?;MUT=F	2213	2159	2682	0.8251305	0.804996271	0.016848674	0.975839	0.976068	150902	154602	0.9937	0.933	0.997	0.9999	1	0.9996	0.9989	0.9998	1	.	P	T	0.372
5	73981270	73981270	T	C	201.3342857	65.19270109	67.28571429	6.8	HEXB	exonic		nonsynonymous	HEXB:NM_0005	Info=CLASS=DM?;MUT=F	1974	1920	2828	0.698019802	0.678925035	0.014453962	0.98143	0.0048059	743	154602	0.9708	0.9923	0.9834	0.9999	0.9789	0.962	0.9725	0.9659	1	.	P	T	0.1
14	88401213	88401213	T	C	687.2618356	63.4421147	84.45714286	15.01369863	GALC;GALC	exonic;splicing		nonsynonymous	GALC:NM_0012	Info=CLASS=DM?;MUT=F	2770	2681	3920	0.706632653	0.683928571	0.017251405	0.940695	0.961411	148636	154602	0.9828	0.8607	0.9918	0.9375	1	0.9996	0.9922	0.9975	1	B	P	T	0.001
X	31496350	31496350	C	T	2071.16548	65.48	329.0420168	16.71717172	DMD	exonic		nonsynonymous	DMD:NM_00401	Info=CLASS=DM?;MUT=F	4192	4006	7105	0.590007037	0.56382829	0.018228146	0.881854	0.0006662	103	154602	0.9045	0.9888	0.7877	0.9234	0.9421	0.9459	0.93	0.7103	0.624	B	P	T	17.1
19	44047826	44047826	T	A	3103.967895	79.98920765	334.5789474	3.150273224	XRCC1;XRCC1	exonic;splicing		nonsynonymous	XRCC1:NM_006	Info=CLASS=DM?;MUT=F	2262	2142	2682	0.843400447	0.798657718	0.037243948	1	0.0063	974	154602	1	0.9999	0.9999	1	1	1	1	1					
7	143017807	143017807	G	T	3373.629333	69.34739561	327.9111111	2.995264744	CLCN1	exonic		nonsynonymous	CLCN1:NM_000	Info=CLASS=DM?;MUT=F	2956	2854	4110	0.719221411	0.694403893	0.019008573	0.99361	0.0001035	16	154602	0.9861	0.9959	0.9959	1	0.9828	0.9783	0.9879	0.9982	1	B	P	T	18.83
15	45392075	45392075	G	A	1619.471351	53.3369525	238	10.62478778	DUOX2;DUOX2	exonic;splicing		nonsynonymous	DUOX2:NM_014	Info=CLASS=DM?;MUT=F	1828	1346	2108	0.867172676	0.638519924	0.167944251	0.720248	0.8492	131288	154602	0.8644	0.2553	0.8539	0.9425	0.9273	0.9248	0.9049	0.942	0.043	B	P	T	23
1	169521849	169521849	T	C	304.8651284	48.35294118	138.733945	17.57429719	F5	exonic		synonymous SNV	F5:NM_000130	Info=CLASS=DM?;MUT=F	330	19	4033	0.081824944	0.004711133	0.038647943	0.0830671	0.0844038	13049	154602	0.0862	0.0874	0.0997	0.0627	0.0561	0.0859	0.0727	0.1028					
3	33055721	33055721	A	G	1853.373489	60.54055258	340.8602151	7.772151899	GLB1	exonic		nonsynonymous	GLB1:NM_00113	Info=CLASS=DM?;MUT=F	3716	3576	5748	0.646485734	0.622129436	0.017676768	0.927117	0.941262	145521	154602	0.98	0.7803	0.9877	1	1	0.9986	0.9878	0.9995	0.915	B	P	T	6.854
16	56904587	56904587	C	G	1048.475035	57.23449914	231.1764706	16.08	SLC12A3	exonic		nonsynonymous	SLC12A3:NM_00	Info=CLASS=DM?;MUT=F	3237	3200	4868	0.664954807	0.65735415	0.005660955	0.990615	0.96557	149279	154602	0.9756	0.9929	0.9946	0.9997	0.9631	0.9635	0.9934	0.9916	1	B	P	T	8.224
8	145640411	145640411	A	G	1264.482582	86.1	133.1573082	26	SLC39A4	exonic		nonsynonymous	SLC39A4:NM_01	Info=CLASS=DM?;MUT=F	1899	1890	4456	0.426166966	0.424147217	0.001281686	0.984625	0.953604	147460	154602	0.9678	0.9933	0.9868	0.9997	0.9482	0.9536	0.9754	0.9862					
5	149360877	149360877	T	C	1043.271208	76.87681159	191.6666667	7.846153846	SLC26A2	exonic		nonsynonymous	SLC26A2:NM_00	Info=CLASS=DM?;MUT=F	2316	2301	2815	0.822735346	0.81740675	0.004505858	0	0.94132	145530	154602	0.9928	0.9983	0.9977	1	0.9982	0.9891	0.9956	0.9947	1	B	P	T	0.011
12	122295335	122295335	T	C	856.8832848	77.58003003	281.6909091	8.31736527	HPD;HPD	exonic;splicing		nonsynonymous	HPD:NM_00215	Info=CLASS=DM?;MUT=F	2292	1974	2828	0.810466761	0.698019802	0.086366105	0.876398	0.834562	129025	154602	0.8502	0.9622	0.7197	0.852	0.775	0.8635	0.8524	0.8465	1	.	P	T	1.004
11	88045583	88045583	A	G	2074.957163	77.50639535	346.2117647	12.43333333	CTSC	exonic		nonsynonymous	CTSC:NM_0018	Info=CLASS=DM?;MUT=F	2034	1505	2350	0.865531915	0.640425532	0.165571205	0.930911	0.874187	135151	154602	0.8861	0.9139	0.923	0.9995	0.8931	0.846	0.8767	0.9427	0.734	B	P	T	0.003
7	6026775	6026775	T	C	2031.492707	70.65927655	394.2407407	23.14705882	PMS2	exonic		nonsynonymous	PMS2:NM_0005	Info=CLASS=DM?;MUT=F	2002	1516	2350	0.851914894	0.645106383	0.152638191	0.883187	0.831263	128515	154602	0.8514	0.9229	0.6563	0.9238	0.8432	0.855	0.8381	0.8961	1	B	P	T	0.135
8	143956693	143956693	G	A	3332.4775	40.91666667	348.1666667	10.58549223	CYP11B1	exonic		nonsynonymous	CYP11B1:NM_00	Info=CLASS=DM?;MUT=A	229	13	3459	0.066204105	0.003758312	0.03128168	0	0.0669396	10349	154602	0.0739	0.0462	0.1282	0.3965	0.0284	0.0185	0.0803	0.1399	1	B	P	T	0.034
6	134210600	134210600	G	T	3301.72	29.46153846	350	13.96923077	TCF21	exonic		nonsynonymous	TCF21:NM_0032	Info=CLASS=DM?;MUT=A	78	4	3351	0.023276634	0.001193674	0.011048074	0.0595048	0.0166233	2570	154602	0.0172	0.1778	0.0092	0.002	0	0.0011	0.0143	0.0017	0.027	B	P	T	14.3
9	135777121	135777121	A	G	1908.086512	86.34883721	324.5214106	8.228323699	TSC1	intronic				Info=CLASS=DM?;MUT=A	627	64	6221	0.100787655	0.010287735	0.045483923	0.17492	0.145593	22509	154602	0.1481	0.348	0.0898	0.0863	0.1176	0.1488	0.1319	0.1055					
11	31815362	31815362	G	A	1018.724675	64.22077922	285.6717949	9.105263158	PAX6	splicing	NM_001604:exon11:c.808-12C>T,NM_000280:exon			Info=CLASS=DM?;MUT=A	735	95	3944	0.186359026	0.024087221	0.082124984	0.0904553	0.0753354	11647	154602	0.0779	0.0398	0.2073	0.1936	0.0554	0.0518	0.0642	0.066					
11	118962804	118962804	A	G	2201.35	74.14285714	283.258427	13.54857143	HMBS	intronic				Info=CLASS=DM?;MUT=A	226	23	3958	0.057099545	0.005811016	0.025718992	0.140176	0.0382725	5917	154602	0.0394	0.4238	0.0188	0	0	0.0021	0.011	0.0007					
3	10183876	10183876	G	C	1012.5	5.75	211.8125	3.5	VHL	splicing	NM_000551:exon1:c.340+5G>C,NM_198156:exon1			Info=CLASS=DM?;MUT=A	17	1	[illegible]	[illegible]	[illegible]	[illegible]	[illegible]	[illegible]	1525	154602	0.0145	0.0005	0.0296	0.025	0	0.0006	0.0153	0.0577					
8	41526116	41526116	G	A	2182.095789	35.84210526	357.6410256	8.314814815	ANK1	intronic				Info=CLASS=DM?;MUT=A	218	21	[illegible]	[illegible]	[illegible]	[illegible]	[illegible]	[illegible]	535	154602	0.0039	0.0006	0.0028	0	0.0003	0.0039	0.0084	0.0107					
10	43606650	43606650	C	T	1564.573333	75	341.3333333	11.38461538	RET	splicing	NM_020630:exon7:c.1264-5C>T,NM_020975:exon7:			Info=CLASS=DM?;MUT=A	26	1	[illegible]	[illegible]	[illegible]	[illegible]	[illegible]	[illegible]	1462	154602	0.0099	0.0983	0.0099	0	0.0003	0.0009	0.0055	0.0002					
15	72643444	72643444	A	C	326.4568857	202	152.5714286	14.66666667	HEXA	intronic				Info=CLASS=DM?;MUT=A	71	2	[illegible]	[illegible]	[illegible]	[illegible]	[illegible]	[illegible]	2939	154602	0.0197	0.0042	0.0066	0.0004	0.0084	0.0214	0.0146	0.0463					
13	113771917	113771917	A	G	1.003527586	180	5.310344828	14.5862069	F7	splicing	NM_019616:exon7:c.739+7A>G,NM_000131:exon8:			Info=CLASS=DM?;MUT=A	27	1	[illegible]	[illegible]	[illegible]	[illegible]	[illegible]	[illegible]	363	154602	0.012	0.0039	0.0073	0.0001	0.0126	0.0179	0.0177	0.0023					
18	55247454	55247454	G	A	1879.518671	63.96202532	328.3324468	5.707610147	FECH	intronic				Info=CLASS=DM?;MUT=A	1031	176	[illegible]	[illegible]	[illegible]	[illegible]	[illegible]	[illegible]	36805	154602	0.2467	0.4468	0.4373	0.3093	0.2329	0.1736	0.2174	0.2577					
2	56098272	56098272	G	A	364.2953521	60.54545455	178.0422535	13.2183908	EFEMP1	splicing	NM_001039349:exon9:c.1001-14C>T,NM_00103934			Info=CLASS=DM?;MUT=A	298	27	[illegible]	[illegible]	[illegible]	[illegible]	[illegible]	[illegible]	8237	154602	0.0534	0.0391	0.0327	0.0005	0.0636	0.0698	0.0694	0.0331					
16	84208329	84208329	G	A	791.3875	111	335.6111111	22.16393443	DNAAF1	splicing	NM_178452:exon10:c.1698+1G>A			Info=CLASS=DM?;MUT=A	60	3	[illegible]	[illegible]	[illegible]	[illegible]	[illegible]	[illegible]	46	154602	0.0003	0.0000961	0.0004	0	0	0.0005	0	0	.	.	N	.	11.68
20	18507194	18507194	G	A	1211.935	182	274.826087	9.535714286	SEC23B	splicing	NM_032985:exon8:c.960+19G>A,NM_006363:exon8			Info=CLASS=DM?;MUT=A	62	4	[illegible]	[illegible]	[illegible]	[illegible]	[illegible]	[illegible]	850	154602	0.006	0.0009	0.0044	0	0.0026	0.0045	0.0088	0.0211					
4	187115777	187115777	G	C	1217.274423	92.28846154	288.6293103	14.6635514	CYP4V2	splicing	NM_207352:exon2:c.327+11G>C			Info=CLASS=DM?;MUT=A	493	53	[illegible]	[illegible]	[illegible]	[illegible]	[illegible]	[illegible]	8166	154602	0.0525	0.0159	0.0402	0	0.0411	0.0766	0.0686	0.0173					
6	160968968	160968968	G	A	2291.52625	57.875	339.7142857	6.8671875	LPA;LPA	exonic;splicing		synonymous SNV	LPA:NM_005577	Info=CLASS=DM?;MUT=A	175	11	[illegible]	[illegible]	[illegible]	[illegible]	[illegible]	[illegible]	1335	154602	0.0089	0.0623	0.01	0	0	0.0049	0.0144	0.0004					
1	94487354	94487354	G	A	233.0248787	74.24101796	63.09248555	18.47502904	ABCA4	intronic				Info=CLASS=DM?;MUT=A	1535	680	[illegible]	[illegible]	[illegible]	[illegible]	[illegible]	[illegible]	[illegible]	[illegible]	[illegible]	[illegible]	[illegible]	[illegible]	[illegible]	[illegible]	[illegible]	[illegible]	[illegible]	[illegible]	[illegible]	[illegible]	[illegible]
16	89815049	89815049	T	C	1859.051818	117.6363636	296.3043478	11.21153846	FANCA	splicing	NM_000135:exon33:c.3348+18A>G,NM_001286167:			Info=CLASS=DM?;MUT=A	117	10	[illegible]	[illegible]	[illegible]	[illegible]	[illegible]	[illegible]	[illegible]	[illegible]	[illegible]	[illegible]	[illegible]	[illegible]	[illegible]	[illegible]	[illegible]	[illegible]	[illegible]	[illegible]	[illegible]	[illegible]	[illegible]
18	11876613	11876613	T	G	499.5842857	119.0952381	111.4316547	17.30670927	GNAL	splicing	NM_182978:exon11:c.1163-7T>G,NM_001142339:e			Info=CLASS=DM?;MUT=A	405	25	[illegible]	[illegible]	[illegible]	[illegible]	[illegible]	[illegible]	[illegible]	[illegible]	[illegible]	[illegible]	[illegible]	[illegible]	[illegible]	[illegible]	[illegible]	[illegible]	[illegible]	[illegible]	[illegible]	[illegible]	[illegible]
11	47357416	47357416	G	A	490.148	65.6	65.6	10.19387755	MYBPC3	splicing	NM_000256:exon25:c.2737+12C>T			Info=CLASS=DM?;MUT=A	105	4	[illegible]	[illegible]	[illegible]	[illegible]	[illegible]	[illegible]	[illegible]	[illegible]	[illegible]	[illegible]	[illegible]	[illegible]	[illegible]	[illegible]	[illegible]	[illegible]	[illegible]	[illegible]	[illegible]	[illegible]	[illegible]
12	103306550	103306550	A	G	1070.587595	75.89873418	278.425	17.46300306	PAH	splicing	NM_000277:exon2:c.168+19T>C			Info=CLASS=DM?;MUT=A	658	95	[illegible]	[illegible]	[illegible]	[illegible]	[illegible]	[illegible]	[illegible]	[illegible]	[illegible]	[illegible]	[illegible]	[illegible]	[illegible]	[illegible]	[illegible]	[illegible]	[illegible]	[illegible]	[illegible]	[illegible]	[illegible]
8	110472066	110472066	G	C	657.9533333	15.7	91.4	7.106666667	PKHD1L1	splicing	NM_177531:exon47:c.7246+1G>C			Info=CLASS=DM?;MUT=A	67	3	[illegible]	[illegible]	[illegible]	[illegible]	[illegible]	[illegible]	[illegible]	[illegible]	[illegible]	[illegible]	[illegible]	[illegible]	[illegible]	[illegible]	[illegible]	[illegible]	[illegible]	[illegible]	[illegible]	[illegible]	[illegible]
7	20666235	20666235	G	C	192.8713526	148	93.75438596	27.21052632	ABCB5	splicing	NM_001163941:exon3:c.108+1G>C			Info=CLASS=DM?;MUT=A	46	1	[illegible]	[illegible]	[illegible]	[illegible]	[illegible]	[illegible]	[illegible]	[illegible]	[illegible]	[illegible]	[illegible]	[illegible]	[illegible]	[illegible]	[illegible]	[illegible]	[illegible]	[illegible]	[illegible]	[illegible]	[illegible]
2	138030234	138030234	C	T	1658.604	70.34507042	180.5774648	12.13849765	THSD7B	splicing	NM_001316349:exon11:c.2396+2C>T			Info=CLASS=DM?;MUT=A	389	41	[illegible]	[illegible]	[illegible]	[illegible]	[illegible]	[illegible]	[illegible]	[illegible]	[illegible]	[illegible]	[illegible]	[illegible]	[illegible]	[illegible]	[illegible]	[illegible]	[illegible]	[illegible]	[illegible]	[illegible]	[illegible]
[illegible]	[illegible]	[illegible]	[illegible]	[illegible]	[illegible]	[illegible]	[illegible]	[illegible]	[illegible]	[illegible]	[illegible]	[illegible]	[illegible]	[illegible]	[illegible]	[illegible]	[illegible]	[illegible]	[illegible]	[illegible]	[illegible]	[illegible]	[illegible]	[illegible]	[illegible]	[illegible]	[illegible]	[illegible]	[illegible]	[illegible]	[illegible]	[illegible]	[illegible]	[illegible]	[illegible]	[illegible]	[illegible]
