# Supplementary material for: Revisiting the morbid genome of Mendelian disorders
Source: Genome Biol. 2016 Nov 24;17:235. doi: 10.1186/s13059-016-1102-1 (PMC5123336; doi:10.1186/s13059-016-1102-1)
Supplement: Additional file 6: Table S6. — Reclassified ClinVar variants based on MAF of 0.01 threshold and lack of phenotype in homozygotes. (PDF 285 kb) [file 13059_2016_1102_MOESM6_ESM.pdf]

Table S6. Reclassified ClinVar variants based on MAF of 0.01 threshold and lack of phenotype in homozygotes

Column titled 1000G includes frequencies in 1000G, columns titled "KAVIAR_AF, KAVIAR_AC" include allele frequencies and counts in KAVIAR DB, column titled "EXAC_ALL" includes average EXAC frquency over different populations, and subsequent EXAC columns include frequencies for each popoulation, columns titled "CLNSDB" to "CLNSIG" includes ClinVar records where "CLINSIG" includes variant class in ClinVar, column titled "SGP_screene" includes number of screenees for that variant from gene panels and exomes, "SGP_C" includes number of occurrences of the valiant, "SGP_C_hom" includes occurences as hom, columns titled "SGP_Freq" and "SGP_Freq_hom" include frequency for all and homozygous ones, "SGP_AF_Strict" includes computation of allele frequency using formula given in the paper, columns titled "SIFT", "PolyPhen", "MutationTaster", "MetaSVM", and "cadd phred" include pahogeneicity predictions using respective tools. Columns titled "AvgQIon" and "AvgQIllum" include average quality scores for Ion and Illumina, and columns titled "AvgIonDepth" and "AvgIlluDepth" include for Ion and Illumina respectively.

chromosome	start pos	end pos	reference	observed	AvgQION	AvgQIllum	AvgIonDepth	AvgIlluDepth	refGene gene	refGene function	refGene splice i	refGene exonic	refGene AA cha	SGP_C	SGP_C_hom	SGP_Screenee	SGPFreq	SGPFreqHom	SGP_AF_Strict	1000G all	Kaviar_AF	Kaviar_AC	Kaviar_AN	CLNDSDB	CLNACC	CLNDBN	CLNDSDBID	CLINSIG	ExAC_ALL	ExAC_AFR	ExAC_AMR	ExAC_EAS	ExAC_FIN	ExAC_NFE	ExAC_OTH	ExAC_SAS	SIFT	PolyPhen 2 HDI	LJB MutationTa	MetaSVM_pred	CADD_phred
17	78083726	78083726	A	G	579.1511329	85.11900161	144.377551	17.11143695	GAA	splicing	NM_000152:exon9:c.1327-18A>G,NM_001079804:e			2094	1630	4627	0.452561055	0.352280095	0.060860441	0.711661	0.720638	111412	154602	MedGen|MedGen	RCV000078156.	not_specified|not	CN169374|CN22	Likely pathogenic	0.7312	0.7012	0.4984	0.6277	0.7962	0.7619	0.7607	0.8174					
8	11614575	11614575	A	G	408.8707421	53.31931818	195.7873303	7.278118609	GATA4;GATA4	exonic		nonsynonymous	GATA4:NM_001	709	105	3541	0.200225925	0.02965264	0.086570159	0.0429313	0.0944554	14603	154602	MedGen|MedGen	RCV000037322.	not_specified|not	CN169374|CN22	Likely pathogenic	0.0962	0.0204	0.0407	0.0006	0.1405	0.1354	0.1247	0.0556	0.566	B	P	T	9.418
2	31570376	31570376	T	C	1733.601132	71.35581761	301.0877863	6.608828006	XDH	splicing	NM_000379:exon29:c.3276+12A>G			2188	1424	3517	0.622121126	0.404890532	0.136185383	0.777356	0.76554	118354	154602	MedGen	RCV000171492.1	not_provided	CN221809	Likely pathogenic	0.7785	0.7292	0.7451	0.8529	0.7578	0.7708	0.7852	0.8329					
19	13010520	13010520	A	G	1354.657181	54.3904048	253.6197917	5.663033606	GCDH;GCDH	exonic		nonsynonymous	GCDH:NM_0139	1742	661	2828	0.615983027	0.233734088	0.216416416	0.720447	0.600413	92825	154602	MedGen:OMIM:C	RCV000190518.1	Glutaric_aciduria	C0268595:23167	Likely pathogenic	0.613	0.8249	0.7161	0.7855	0.4553	0.5435	0.5511	0.671					
18	44109033	44109033	T	C	3007.861588	0	353.3356164	0	LOXHD1	intronic				1097	586	2604	0.421274962	0.217357911	0.114390349	0.620208	0.686876	17878	26028	MedGen	RCV000171270.1	not_provided	CN221809	Likely pathogenic													
6	151936677	151936677	G	A	185.854772	85.3134715	29.37046632	18.55420466	CCDC170	exonic		nonsynonymous	CCDC170:NM_0	1103	315	2000	0.5515	0.1575	0.213839891	0.349441	0.307053	47471	154602	MedGen:OMIM	RCV000143990.1	Estrogen_resista	C1851467:615363	Likely pathogenic	0.3172	0.5003	0.191	0.3218	0.1861	0.3212	0.3056	0.3314	0.356	B	P	T	11.74
2	227892720	227892720	C	T	1059.468034	66.56415584	379.0434783	10.41296296	COL4A4;COL4A4	exonic		nonsynonymous	COL4A4:NM_00	1550	407	3293	0.470695415	0.123595506	0.184981389	0.493011	0.448804	69386	154602	MedGen	RCV000171504.1	not_provided	CN221809	Likely pathogenic	0.4879	0.5479	0.5562	0.424	0.5097	0.4513	0.4898	0.5698	0.58	B	P	T	14.35
1	9307230	9307230	T	A	508.0656729	10.92411215	127.1741935	3.598130841	H6PD	intronic				605	133	2936	0.20606267	0.045299728	0.082244293	0.250799	0.0473862	7326	154602	MedGen	RCV000171451.1	not_provided	CN221809	Likely pathogenic													
13	52971718	52971718	G	C	1563.847368	81.44736842	196.0481928	17.74698795	THSD1	exonic		nonsynonymous	THSD1:NM_018	309	30	2000	0.1545	0.015	0.07077078	0.0533147	0.0496113	7670	154602	MedGen:OMIM:C	RCV000170586.1	Non-immune_hyd	C0455988:23675	Likely pathogenic	0.0504	0.0911	0.022	0.0092	0.0431	0.0515	0.041	0.065	0.399	P	N	T	13.34
17	39975704	39975704	G	T	2482.05	61.99038462	260.75	9.096153846	FKBP10	intronic				97	7	2815	0.03445826	0.002486679	0.016005691	0.00539137	0.0023027	356	154602	MedGen	RCV000171257.1	not_provided	CN221809	Likely pathogenic													
21	43161469	43161469	G	A	683.022	86.2	250	21.08139535	RIPK4	exonic		synonymous SN	RIPK4:NM_0206	109	7	2815	0.038721137	0.002486679	0.018139783	0.00159744	0.0053298	824	154602	MedGen	RCV000171349.1	not_provided	CN221809	Likely pathogenic	0.0056	0.0015	0.0014	0.0002	0.0144	0.008	0.0023	0.0011					
2	233684544	233684544	C	T	222.2293333	59.66666667	98.45714286	6.316455696	GIGYF2;GIGYF2	exonic		nonsynonymous	GIGYF2:NM_001	103	7	3920	0.02627551	0.001785714	0.012255841	0	0.0000259	4	154602	MedGen:OMIM	RCV000198246.1	Parkinson_diseas	C1843211:607688	Likely pathogenic	0.0000261	0	0	0	0	0.0000474	0	0	0.043	D	D	T	23.7
3	100948418	100948418	G	A	3741.623333	62.45238095	394	13.38095238	IMPG2;IMPG2	exonic		nonsynonymous	IMPG2:NM_0162	51	3	3646	0.013987932	0.00082282	0.006585265	0.0171725	0.0057826	894	154602	MedGen	RCV000171365.1	not_provided	CN221809	Likely pathogenic	0.0061	0.0674	0.0037	0	0	0.0002	0.0078	0.00006093	0.088	B	N	T	9.562
1	152275789	152275789	C	T	1787.4	111	357.3043478	24.84444444	FLG	exonic		nonsynonymous	FLG:NM_002016	63	3	3848	0.016372141	0.000779626	0.007799298	0.0155751	0.0040556	627	154602	MedGen	RCV000171158.1	not_provided	CN221809	Likely pathogenic	0.0041	0.0409	0.0013	0.0001	0.0002	0.0007	0.0011	0.0005	0.13	B	N	T	4.081
2	198571223	198571223	A	G	1426.065	111	149.25	31.2125	MARS2	exonic		nonsynonymous	MARS2:NM_138	67	1	2000	0.0335	0.0005	0.016504126	0	0.0001682	26	154602	MedGen	RCV000195863.1	not_provided	CN221809	Likely pathogenic	0.0002	0	0.00008637	0	0	0.0003	0	0.0002	0.001	D	D	T	25.1
[illegible]	[illegible]	[illegible]	[illegible]	[illegible]	[illegible]	[illegible]	[illegible]	[illegible]	[illegible]	[illegible]	[illegible]	[illegible]	[illegible]	[illegible]	[illegible]	[illegible]	[illegible]	[illegible]	[illegible]	[illegible]	[illegible]	[illegible]	[illegible]	[illegible]	[illegible]	[illegible]	[illegible]	[illegible]	[illegible]	[illegible]	[illegible]	[illegible]	[illegible]	[illegible]	[illegible]	[illegible]	[illegible]	[illegible]	[illegible]	[illegible]	[illegible]

Table06_Reclassified_ClinVar_vs
