# Supplementary material for: Revisiting the morbid genome of Mendelian disorders
Source: Genome Biol. 2016 Nov 24;17:235. doi: 10.1186/s13059-016-1102-1 (PMC5123336; doi:10.1186/s13059-016-1102-1)
Supplement: Additional file 9: Table S9. — Reclassified BRCA variants based on lack of phenotype in homozygotes. (PDF 125 kb) [file 13059_2016_1102_MOESM9_ESM.pdf]

Table S9. Reclassified *BRCA* variants based on lack of phenotype in homozygotes

Column titled "SGP_screene" includes number of screenees for that variant from gene panels and exomes, "SGP_C" includes number of occurrences of the vatiant, "SGP_C_hom" includes occurrences as hom, columns titled "SGP_Freq" and "SGP_Freq_hom" include frequency for all and homozygous ones, "SGP_AF_Strict" includes computation of allele frequency using formula given in the paper, columns titled "SIFT", "PolyPhen", "MutationTaster", "MetaSVM", and "cadd phred" include pahogeneicity predictions using respective tools. Columns titled "AvgQIon"" and "AvgQillum" include average quality scores for Ion and Illumina, and columns titled "AvgIonDepth" and "AvgIlluDepth" include average depth for Ion and Illumina respectively.

chromosome	start pos	end pos	reference	observed	AvgQION	AvgQIllum	AvgIonDepth	AvgIlluDepth	refGene splice i	refGene exonic f	refGene AA chai	Pathogenicity_d	id	Gene_Symbo	Reference_Sequ	HGVS_cDNA	HGVS_Protein	SGP_C	SGP_C_hom	Freq	SGP_AF_Stric	SGP_Screene	SIFT	PolyPhen 2 HDI\	LJB MutationTa	MetaSVM_pred	CADD_phred
chr17	4125609	4125610	AAAAAAAAGAA	-	61.152	567	6.6	13.4				Not Yet Classified	11976	BRCA1	NM_007294.3	NM_007294.3:c.4	NP_009225.1:p.	6	1	0.003	0.00125031	2379					
chr13	3293737	3293737	A	G	2961.65	196	316	8		nonsynonymous	BRCA2:NM_000	Not Yet Classified	8744	BRCA2	NM_000059.3	NM_000059.3:c.8	NP_000050.2:p.(Asp2680Gl	1	1	0.0005	0	2379	0.001	D	D	D	31
chr13	3291413	3291413	T	G	483.905	222	196.5	12.1176470		nonsynonymous	BRCA2:NM_000	Not Yet Classified	9799	BRCA2	NM_000059.3	NM_000059.3:c.5	NP_000050.2:p.(Asn1880Ly	12	1	0.006	0.00275068	2379	0.007	B	N	T	14.64
chr13	3291327	3291327	A	C	330.101833	174	165	8.5		nonsynonymous	BRCA2:NM_000	Not Yet Classified	7584	BRCA2	NM_000059.3	NM_000059.3:c.4	NP_000050.2:p.(Glu1593As	10	1	0.005	0.00225056	2379	0.14	B	N	T	17.41
chr13	3294479	3294479	G	A	552.95	50	258.666666	8				Not Yet Classified	495	BRCA2	NM_000059.3	NM_000059.3:c.8	NP_000050.2:p.	5	1	0.0025	0.0010002	2379					
chr13	3289072	3289072	A	C	442.39	15.6	325	8				Not Yet Classified	2080	BRCA2	NM_000059.3	NM_000059.3:c.6	NP_000050.2:p.	2	1	0.001	0.00025006	2379					
chr13	3290052	3290052	T	C	3182.08	155	332	8				Not Yet Classified	2501	BRCA2	NM_000059.3	NM_000059.3:c.5	NP_000050.2:p.	2	1	0.001	0.00025006	2379					
chr13	3297275	3297275	T	C	430.7057	188	180.25	18.6842105		nonsynonymous	BRCA2:NM_000	Not Yet Classified	2532	BRCA2	NM_000059.3	NM_000059.3:c.1	NP_000050.2:p.(Ser3368Pr	15	1	0.0075	0.00350087	2379	0.01	B	N	T	6.705
chr13	3293686	3293686	C	A	2227.99	21.259512	231	7.4390243				Not Yet Classified	4785	BRCA2	NM_000059.3	NM_000059.3:c.7	NP_000050.2:p.	34	1	0.017	0.00825206	2379					
chr13	3289334	3289334	A	G	1614.57	148	270	40		synonymous SN	BRCA2:NM_000	Not Yet Classified	2758	BRCA2	NM_000059.3	NM_000059.3:c.1	NP_000050.2:p.(=	4	1	0.002	0.00075018	2379					
chr13	3294477	3294477	G	A	153.47	27	88.6666666	8				Not Yet Classified	4833	BRCA2	NM_000059.3	NM_000059.3:c.8	NP_000050.2:p.	12	1	0.006	0.00275068	2379					
chr13	3292943	3292943	G	A	602.16	107.9	528	10.6363636	NM_000059:exon14:c.7435+6G			Not Yet Classified	12550	BRCA2	NM_000059.3	NM_000059.3:c.7	NP_000050.2:p.	9	2	0.0045	0.00175087	2379					
chr13	3294447	3294447	T	C	557.4775	143	387.25	8				Not Yet Classified	7414	BRCA2	NM_000059.3	NM_000059.3:c.8	NP_000050.2:p.	9	2	0.0045	0.00175087	2379					
chr13	3293188	3293188	A	G	163.753333	74	52.9333333	4.33333333		nonsynonymous	BRCA2:NM_000	Not Yet Classified	10908	BRCA2	NM_000059.3	NM_000059.3:c.7	NP_000050.2:p.(Tyr2543Cy	16	3	0.008	0.00325243	2379	0.079	B	D	T	23.7
chr13	3289072	3289072	T	G	992.443818	41.12	302.272727	6.4				Not Yet Classified	11906	BRCA2	NM_000059.3	NM_000059.3:c.6	NP_000050.2:p.	76	4	0.038	0.01801801	2379					
chr13	3289326	3289326	C	T	1110.43463	32.0666666	268.846153	14.48		nonsynonymous	BRCA2:NM_000	Not Yet Classified	10406	BRCA2	NM_000059.3	NM_000059.3:c.1	NP_000050.2:p.(Pro41Le	61	4	0.0305	0.01426426	2379	0.092	B	N	T	6.3
chr17	4125610	4125610	AAG	-	80.997	1191.84615	98.1666666	16.4230769				Not Yet Classified	7281	BRCA1	NM_007294.3	NM_007294.3:c.4	NP_009225.1:p.	101	22	0.0505	0.01985922	2379					
chr13	3292938	3292938	T	C	1083.96242	77.9818181	150.636363	11.4181818		nonsynonymous	BRCA2:NM_000	Not Yet Classified	9742	BRCA2	NM_000059.3	NM_000059.3:c.7	NP_000050.2:p.(=	1867	1821	0.9335	0.02111060	2379	1	.	P	T	0.372
